# Supplementary material for: Laboratory evaluation of a bio-insecticide candidate from tangerine peel extracts against Trialeurodes vaporariorum (Homoptera: Aleyrodidae)
Source: PeerJ. 2024 Mar 19;12:e16885. doi: 10.7717/peerj.16885 (PMC10959105; doi:10.7717/peerj.16885)
Supplement: Supplemental Information 1 [file peerj-12-16885-s001.zip › Data/Apparatus for the extraction and recovery solvents.pdf]

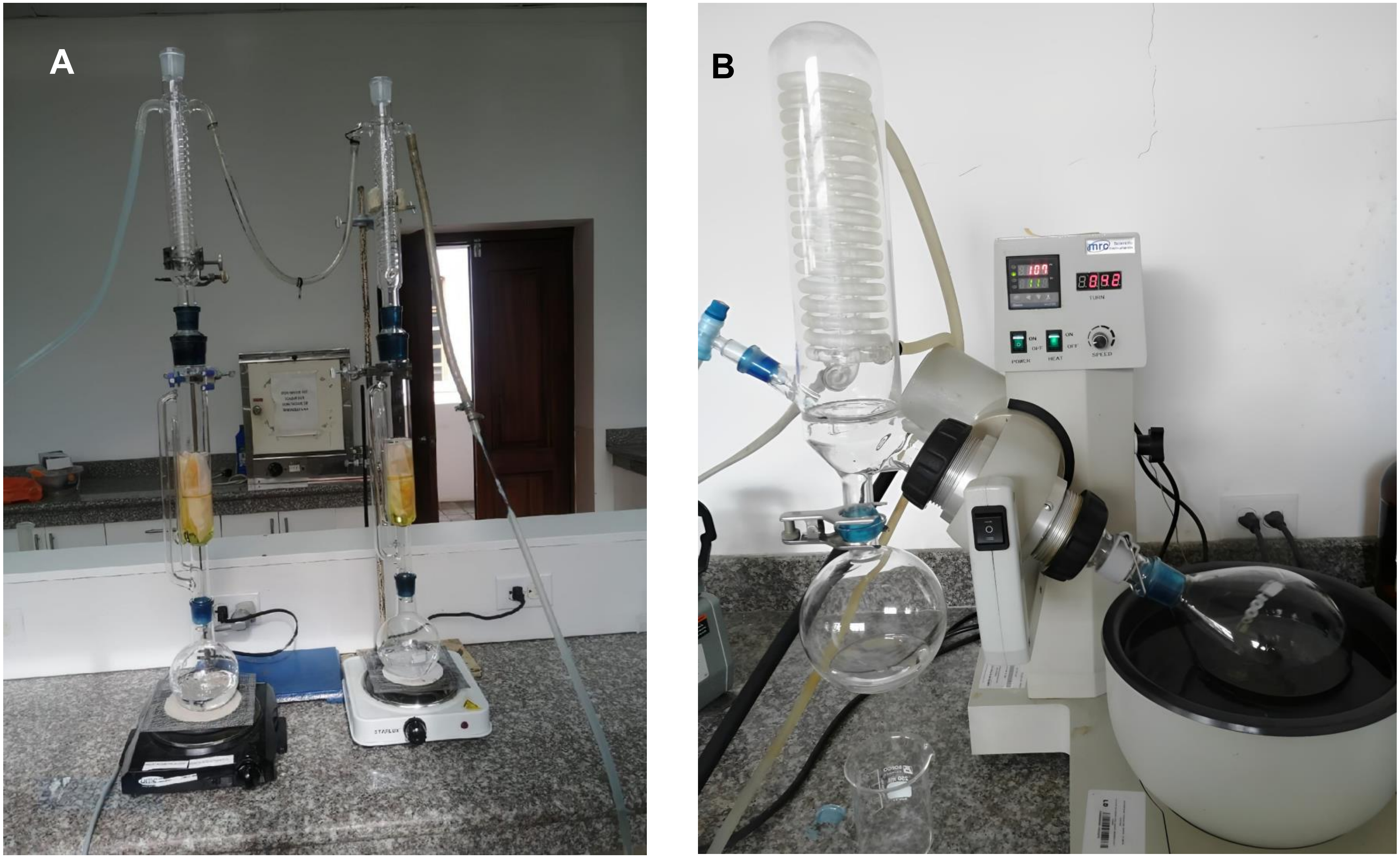

Apparatus for the extraction with solvents of the essential oil of the tangerine (*Citrus reticulata* L.) peel. (A) 250 mL Soxhlet apparatus. (B) Rotary evaporator for the removal of the solvent used.
