# Supplementary material for: Laboratory evaluation of a bio-insecticide candidate from tangerine peel extracts against Trialeurodes vaporariorum (Homoptera: Aleyrodidae)
Source: PeerJ. 2024 Mar 19;12:e16885. doi: 10.7717/peerj.16885 (PMC10959105; doi:10.7717/peerj.16885)
Supplement: Supplemental Information 1 [file peerj-12-16885-s001.zip › Data/Devices for culturing whiteflies and carrying out experiments .pdf]

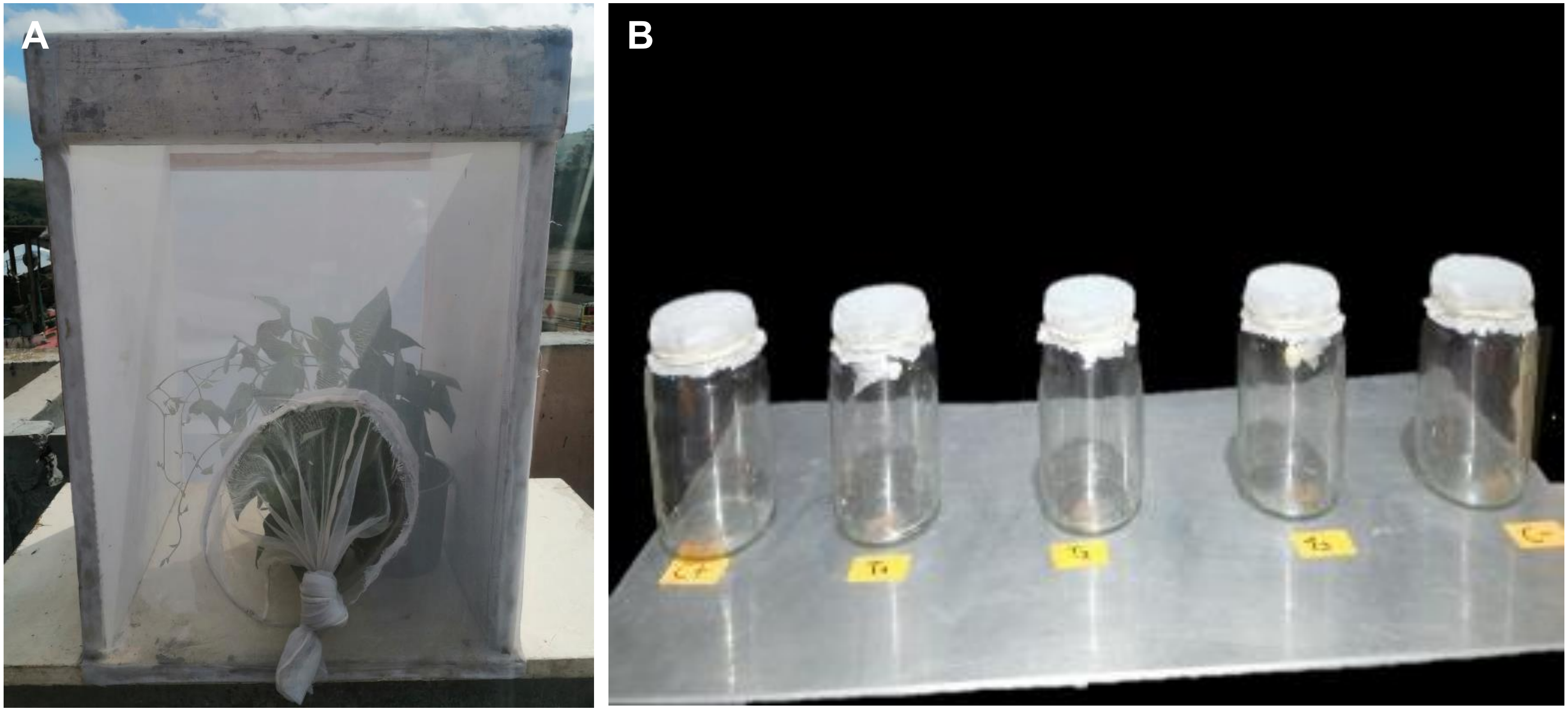

Devices for culturing whiteflies and carrying out experiments to determine the dose-effect relationship of the different treatments. (**A**) An entomological and self-made box with a greenhouse-cultivated tomato plant (*Solanum lycopersicum* L.) infected with greenhouse whiteflies (*Trialeurodes vaporariorum* W.) to collect adult whiteflies; (**B**) The creation an experimental block with ten adult greenhouse whiteflies per 250 mL bottle, made up of the control (negative and positive) and the three treatments with EOEs (T1 – 12.5%, T2 – 25.0%, and T3-33.3% (v/v)).
