# Supplementary material for: Laboratory evaluation of a bio-insecticide candidate from tangerine peel extracts against Trialeurodes vaporariorum (Homoptera: Aleyrodidae)
Source: PeerJ. 2024 Mar 19;12:e16885. doi: 10.7717/peerj.16885 (PMC10959105; doi:10.7717/peerj.16885)

Sample ID:Pais 14-04-22 liquid

Method Name:C:\Users\Public\Documents\Agilent\MicroLab\Methods\HR-400-4000 TRA.a2m

Sample Scans:8

User:QUIMICA

Background Scans:8

Date/Time:04/14/2022 9:28:39 AM

Resolution:4

Range:4000 - 400

System Status:Good

Apodization:Happ-Genzel

File Location:C:\Users\Public\Documents\Agilent\MicroLab\Results\\Pais 14-04-22 liquid_4-14-2022T9-28-39 AM.a2r

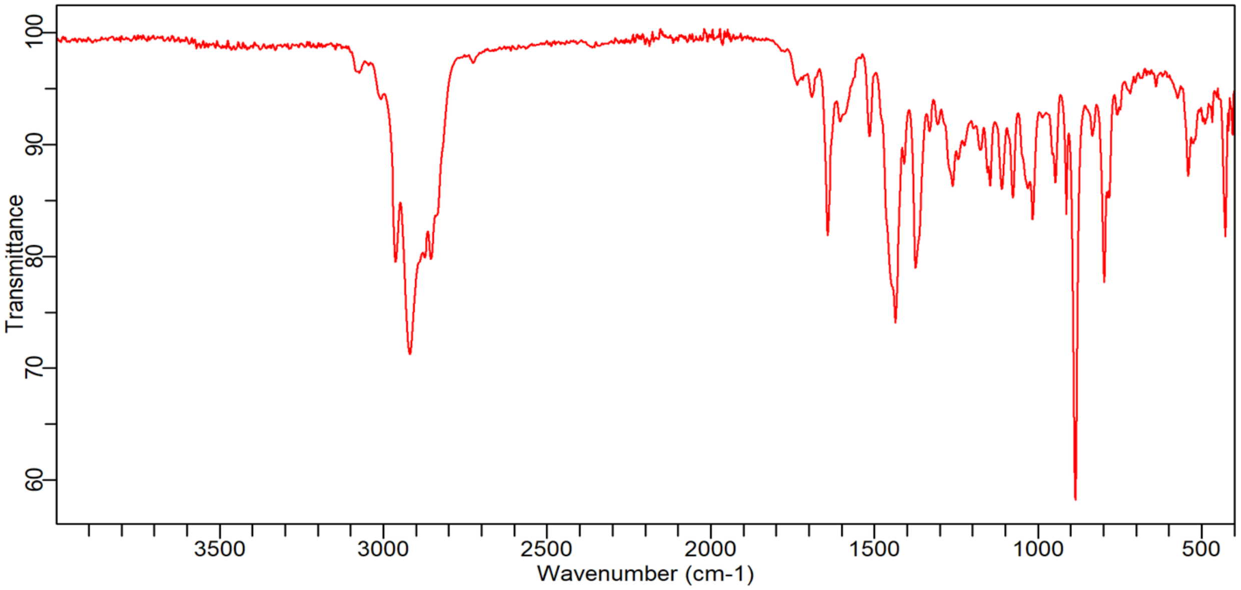

Supplement: Supplemental Information 2 [file peerj-12-16885-s002.zip › FTIRSpectra/HEX_4-14-2022T9-28-39 AM.pdf]
